# Bio-Pathological Markers in the Diagnosis and Therapy of Cancer

**DOI:** 10.3390/cancers12113113

**Published:** 2020-10-25

**Authors:** Giuseppe Broggi, Lucia Salvatorelli

**Affiliations:** Department G.F. Ingrassia, Azienda Ospedaliero-Universitaria “Policlinico-Vittorio Emanuele”, Anatomic Pathology, University of Catania, 95 125 Catania, Italy; lucia.salvatorelli@unict.it

**Keywords:** cancer, prognostic and predictive factors, diagnosis, therapy, immunohistochemical markers, molecular markers, malignant neoplasms

## 1. Diagnostic Markers in Human Cancer

The two medical sciences that mostly deal with the diagnostic approach to human neoplasms in clinical practice are undoubtedly radiology and pathology. The combined radio-pathological approach allows better diagnostic accuracy and enhances the effectiveness of the surgical procedure [1]. While radiology provides a macroscopic “point of view” of tumors, pathology allows the study of the microscopic, cellular details of human neoplasms. Although the interpretation of the morphology of the tumor is still a mandatory step in the pathologist’s routine practice, the introduction of immunohistochemistry (IHC) has undoubtedly represented a turning point, improving the histopathological diagnostic “quality” [2]. Using immunohistochemical antibodies against specific antigens of neoplastic cells, pathologists are often able to identify the cell line of origin even in the case of poorly differentiated tumors [2]. IHC also enables the identification of specific proteins selectively expressed by some types of neoplasms with high sensitivity and specificity rates [2]: a classic example is represented by anti-STAT6 C-terminal antibodies, routinely used in diagnostic practice as surrogate for the NAB2-STA6 fusion, present in almost all cases of solitary fibrous tumor (SFT) [3]. In the last decades, the diagnostic utility of IHC has been integrated and enhanced by the development of methods, such as Western blot, fluorescence in situ hybridization (FISH), real-time polymerase chain reaction (rt-PCR), and next-generation sequencing (NGS), able to investigate human neoplasms at a molecular level, identifying the specific mutational status of tumors. In particular, some “entity-defining” mutations have been introduced in different areas of human oncology: The fact that in neuro-oncology a diagnosis of “oligodendroglioma” may be rendered only in the presence of the Isocytrate Dehydrogenase (IDH) 1/2 mutation and 1p-19q codeletion is emblematic [4]. Such methods, mainly due to their costs, cannot replace IHC, but must be integrated with it, representing a second step in the diagnostic approach to human tumors. In this regard, the possibility of combining an easily accessible technique such as IHC with the search for microRNAs (miRNA) by in situ hybridization (ISH) represents one of the most interesting alternatives [5]; this combined method can be performed on paraffin-embedded and formalin-fixed tissue and allows miRNAs amplification to be evaluated, determining both their spatial tissue distribution and cellular/subcellular localization, based on the correlation with immunohistochemical stains [5]; currently the other PCR-based miRNAs extraction techniques do not allow such results to be obtained.

## 2. Prognostic and Predictive Markers of Therapeutic Response in Human Cancer

In human oncology a prognostic factor is a protein, gene or mutation that confers a better or worse prognosis to a tumor; the usefulness of identifying certain prognostic factors is represented by the possibility to prognostically subclassify patients affected by a specific neoplasm in terms of overall survival (OS) and disease-free survival (DFS) [6]. Many prognostic factors also have a predictive value: their high or low expression makes that kind of tumor more or less sensitive to specific therapies. Accordingly, based on their evaluation, it has been demonstrated that a therapeutic “customization” may be performed with decisive impact on OS and DFS. Both IHC and molecular methods allow the detection of prognostic/predictive factor in human cancer. One of the major application fields of these findings is undoubtedly breast cancer: the identification of factors with both prognostic and predictive value, easily detectable by IHC, such as expression of estrogen and progesterone receptors (ER, PgR), c-erbB2 (HER-2) overexpression and proliferative index, led to a progressive abandonment of the old morphological classification of breast cancer for the new “molecular” one. Based on the different expression levels of ER, PgR, c-erbB2 and proliferative index, a subdivision into “Luminal A”, “Luminal B”, “HER-2 +” and “basal-like” breast cancer is currently widely used [7]. Particularly, the determination of c-erbB2 status by IHC and FISH (in cases with non- conclusive immunohistochemical results) has the highest clinical impact as it selects a subgroup of patients with poorer prognosis but who can benefit from therapy with anti-HER2 monoclonal antibody (trastuzumab) [8]. Central nervous system tumors represent another field in which decisive progress has been made through the identification of tumor-related prognostic/predictive markers. The most relevant molecular signature of World Health Organization (WHO) grade IV glioblastomas (GBMs) is promoter methylation of the gene encoding for O^6^-methylguanine DNA methyltransferase (MGMT), located at chromosome 10q26 and coding for an ubiquitously expressed suicide DNA repair enzyme which induces the removal of alkyl adducts from the O^6^-guanine [9]. Inactivation of MGMT leads to cell death caused by DNA breaks; it also has a protective role on tumor cells from the lethal effects of chemotherapy with alkylating agents such as temozolomide [9]. About 35–45% of WHO grades III and IV malignant gliomas present methylation of the MGMT promoter that strongly predicts a better response to alkylating agents, representing a powerful and independent prognostic/predictive factor of longer OS and DFS [9].

## 3. Future Perspectives

The search of bio-pathological markers with diagnostic and prognostic/predictive role still represents one of the most important perspectives of global scientific research. In recent years most efforts have been made to identify new bio-markers of particularly rare forms of cancer, such as uveal melanoma (UM). It has been demonstrated that inactivating mutations of BAP-1 gene, easily detectable by the immunohistochemical loss of BAP-1, select a subgroup of UMs with more aggressive clinical behavior and higher metastatic potential [10]. Moreover, the differential immunohistochemical expression of other proteins, including ADAM-10, ABCB5, SPANX-C, and MacroH2A1 [6,11,12,13], has been found to have potential prognostic significance in UM. Programmed Death-1 (PD-1) and Programmed Death Ligand-1 (PDL-1) represent the prototypes of relatively new prognostic/predictive markers, whose role is being investigated in several human neoplasms, including non-small-cell lung cancer (NSCLC), as they identify a subset of patients potentially susceptible to therapy with PD-1 and PDL-1 inhibitors [14].

However, despite the many advances made, very little is known about the true biology of human cancer. This is encouraging the scientific community to search for additional bio-pathological markers in order to better know the cancer biology, enhance the diagnostic accuracy, and provide further potential prognostic information with an impact on patient treatment.
